# Virtual Reality Exercise for Anxiety and Depression: A Preliminary Review of Current Research in an Emerging Field

**DOI:** 10.3390/jcm7030042

**Published:** 2018-03-04

**Authors:** Nan Zeng, Zachary Pope, Jung Eun Lee, Zan Gao

**Affiliations:** 1 School of Kinesiology, University of Minnesota Twin Cities, Minneapolis, MN 55455, USA; zengx185@umn.edu (N.Z.), popex157@umn.edu (Z.P.); 2 Department of Applied Human Sciences, University of Minnesota Duluth, Duluth, MN 55812, USA; junelee@d.umn.edu

**Keywords:** anxiety, depression, exercise, mental health, virtual reality

## Abstract

Objective: Although current evidence supports the use of virtual reality (VR) in the treatment of mental disorders, it is unknown whether VR exercise would be beneficial to mental health. This review synthesized literature concerning the effect of VR exercise on anxiety and depression among various populations. Methods: Ten electronic databases were searched for studies on this topic from January 2000 through October 2017. Studies were eligible if the article: (1) was peer-reviewed; (2) was published in English; and (3) used quantitative measures in assessing anxiety- and depression-related outcomes. Results: A total of five empirical studies met the eligibility criteria. These studies included two randomized clinical trials, one control trial, and two cross-sectional studies. Four studies reported significant improvements in anxiety- and depression-related measures following VR exercise, including reduced tiredness and tension, in addition to increased energy and enjoyment. Nonetheless, one study failed to support the effectiveness of VR exercise over traditional exercise alone on depressive symptoms. Conclusions: Findings favor VR exercise in alleviating anxiety and depression symptomology. However, existing evidence is insufficient to support the advantages of VR exercise as a standalone treatment over traditional therapy in the alleviation of anxiety and depression given the paucity of studies, small sample sizes, and lack of high-quality research designs. Future studies may build upon these limitations to discern the optimal manner by which to employ VR exercise in clinical settings.

## 1. Introduction

Anxiety is a psychological disorder characterized by worried thoughts, feelings of tension, and physical changes such as increased blood pressure [1]. Individuals with anxiety disorders tend to have recurring intrusive thoughts or concerns, as well as specific physical symptoms such as trembling, sweating, a rapid heartbeat, or dizziness [1]. Today, anxiety disorders are the most common mental illness in the U.S., affecting approximately 40 million adults (18.1% of the U.S. population) annually. However, only 36.9% of those suffering from any anxiety disorder receive treatment [2]. Depression is another major psychological disorder. Depressed individuals present not only with poor mood, but also with disturbed sleep or appetite, significant weight loss or gain, loss of interest or pleasure in daily activities, lack of energy, inability to concentrate, feelings of worthlessness, and recurrent thoughts of death or suicide [1]. In 2015, 16.1 million U.S. adults had experienced at least one major depressive episode within the past year (6.7% of all U.S. adults) [2]. Remarkably, while anxiety and depression disorders are different, individuals who develop depression may have experienced an anxiety disorder earlier in life. Indeed, individuals with depression often experience symptomology similar to that characteristic of anxiety disorders, including nervousness, irritability, disturbed sleep or appetite, and poor concentration, among other symptoms [2]. Fortunately, anxiety and depression disorders are treatable, with most individuals able to be helped with the appropriate professional care. Concerted and novel efforts, therefore, must continue to be made to promote mental health among individuals with anxiety and depression through innovative treatment approaches.

Many treatments are available to treat anxiety and depression disorders, including medication, exercise, meditation, and cognitive behavioral therapy. In many cases, these treatments can be tailored to a client to help reduce symptomology of anxiety and/or depression. To date, the application of emerging technology in health promotion has generated substantial public interest. Among the emerging technologies that may potentially aid in the treatment of anxiety and depression, virtual reality (VR) is arguably the most exciting and technologically-advanced. VR is a digital technology that artificially creates sensory experiences—including visual, auditory, touch, and scent stimuli—while allowing the user to manipulate objects within the virtual environment created [3]. Two types of VR exist: (1) immersive VR, which frequently uses head-mounted displays, body-motion sensors, real-time graphics, and advanced interface devices (e.g., specialized helmets) in the simulation of a completely virtual environment for the user; and (2) non-immersive VR, which uses interfaces such as flat-screen televisions/computer screens with associated keyboards, gamepads, and joysticks. Simply stated, immersive VR seeks to envelop the players in a virtual world, making the players feel as though they are “actually there”, whereas the non-immersive VR does not simulate a virtual world to as deep a degree.

Over the past decade, VR applications were widely used for rehabilitation medicine (e.g., stroke, Parkinson’s disease, and developmental disabilities) and behavioral medicine (e.g., phobias, post-traumatic stress disorder, and autism) [4,5,6]. Notably, VR-based treatments for different mental health conditions have observed positive findings. Specifically, VR has been investigated in the treatment of phobias, obesity, chronic pain, and eating disorders [7,8,9,10]. Recently, VR exposure therapy (VRET) has become popular in the treatment of anxiety and depression, with a growing body of literature suggesting that VRET is a successful tool for the treatment of anxiety- and depression-related symptomology [11,12,13,14,15]. Yet, the current literature concerning the effects of VR-based treatments on anxiety and depression primarily focuses on VRET, with VR exercise in the treatment of these conditions being seldom reviewed. VR exercise refers to equipping traditional exercise equipment like bikes and treadmills with VR capabilities. Specifically, some new exercise apparatus has been equipped with integrated sensors that sync with a computer or gaming console and allow the player to engage in strenuous physical exertion on the apparatus while simultaneously engaging in VR gameplay. Researchers and health professionals have documented that combining VR with exercise equipment (a.k.a., VR exercise) may serve to enhance the psychological benefits of exercise and increase the chances of long-term adherence to exercise [16,17]. For example, the latest commercially-available VR exercise apparatus—the VirZoom, a VR exercise bike compatible with most VR headsets (e.g., the Oculus Rift, PlayStation VR, Samsung Gear VR, HTC Vive, etc.)—is presenting researchers and health professionals with the opportunity to implement VR exercise to promote improved health. Thus, given the enjoyable nature of currently-available VR exercise games and the demonstrated positive effects exercise has on anxiety and depression [16,18,19], VR exercise might be viewed as a potentially effective approach to alleviate anxiety- and depression-related symptomology.

As VR exercise is increasing in popularity given the compatibility of VR systems with traditional exercise apparatus such as bikes and treadmills, the potential of VR exercise to be used in the treatment of anxiety and depression is great. Unfortunately, there have been no known comprehensive reviews that have specifically examined the effectiveness of VR exercise on anxiety- and depression-related outcomes. The purpose of this review, therefore, is to systematically evaluate the available evidence concerning the effects of VR exercise on anxiety and depression. In detail, this preliminary review aims to identify, synthesize, and interpret the best available evidence for the use of VR exercise in promoting anxiety- and depression-related outcomes. Findings of this review will help inform scholars and health professionals of the potential benefits VR exercise has in the treatment of anxiety- and depression-related symptomology and where improvements can be made to VR exercise interventions that seek to reduce these symptoms among patients.

## 2. Methods

The Preferred Reporting Items for Systematic Review and Meta-Analysis Protocols (PRISMA-P) 2015 statement was consulted and provided the framework for this review [20].

### 2.1. Information Sources and Search Strategies

The following electronic databases were employed for the literature search: Academic Search Complete, Communication and Mass Media Complete, Education Resources Information Center (ERIC), Google Scholar, Medline, PsycINFO, PubMed, Scopus, SPORTDiscus, and Web of Science. The literature search was conducted independently by the co-authors, with all studies queried placed within a shared research folder. Search terms were discussed among the research team and used in combination: (“virtual reality” OR “head-mounted display”) AND (“exercise” OR “physical activity” OR “sports” OR “bike” OR “treadmill”) AND (“mental illness” OR “mental disorders” OR “mental health” OR “anxiety” OR “depression”).

### 2.2. Eligibility Criteria

The eligibility criteria used to evaluate each study included: (1) published in English between January 2000 through October 2017 as peer-reviewed study; (2) employed the use of immersive VR only (e.g., head-mounted display(s)), with studies of non-immersive VR (e.g., Xbox 360 Kinect and Nintendo Wii) excluded; (3) involved human participants; (4) used quantitative measures in assessing anxiety- and depression-related outcomes; and (5) employed an established study design that allowed for examination of the effect of VR exercise on anxiety- and depression-related outcomes (e.g., randomized controlled trials (RCTs), cohort, and observational studies), meaning case studies were excluded.

### 2.3. Data Extraction

Three reviewers (NZ, ZP, and JL) separately screened the titles of potentially relevant articles. If the reviewers were unable to determine the relevance of an article to the topic, then the abstract was reviewed. Data extraction was completed by one reviewer (NZ) and checked by another (ZP) for accuracy. All potential articles were downloaded as full text and stored in a shared folder, after which three authors (NZ, ZP, and JL) reviewed each article independently to ensure that only relevant entries were included. A list of published articles on the topic was then created in a Microsoft Excel spreadsheet (Microsoft Corporation, Redmond, WA, USA). The following information was extracted: (1) year of publication and country of origin, (2) methodological details (e.g., study design, sample characteristics, study duration, VR exposure, anxiety- and depression-related outcomes, and instruments), and (3) key findings concerning the effectiveness of VR exercise on anxiety- and depression-related outcomes. Finally, relevant studies were further identified through cross-referencing the bibliographies of selected articles. Notably, reviewers were not blinded to the authors or journals, and no attempts were made to contact investigators or correspondents of the original studies to acquire any information missing from the included articles.

### 2.4. Risk of Bias in Individual Studies

To assess the risk of bias in each study, two reviewers (NZ, ZP) independently rated each study using an 8-item quality assessment tool (see Table 1) employed in previous literature [21,22,23]. Each item within each study was rated as “positive” (when the item was explicitly described and present) or “negative” (when the item was inadequately described or absent). Two reviewers (NZ, ZP) separately scored each article to ensure reliable scoring of the quality assessment. When disagreements occurred between the two reviewers, unresolved differences were evaluated by a third reviewer (JL). In particular, Items 1, 3, 4, and 6 in Table 1 were deemed the most important, as these items had greater potential to significantly affect the research findings. Of note, the final score for each study was calculated by summing up the all “positive” ratings. A study was considered “high-quality and at low risk of bias” when scored above the median score of 4.5 following the scoring of all studies. Studies below the median score were considered “low-quality and at high risk of bias”.

## 3. Results

### 3.1. Study Selection

**Figure 1:**
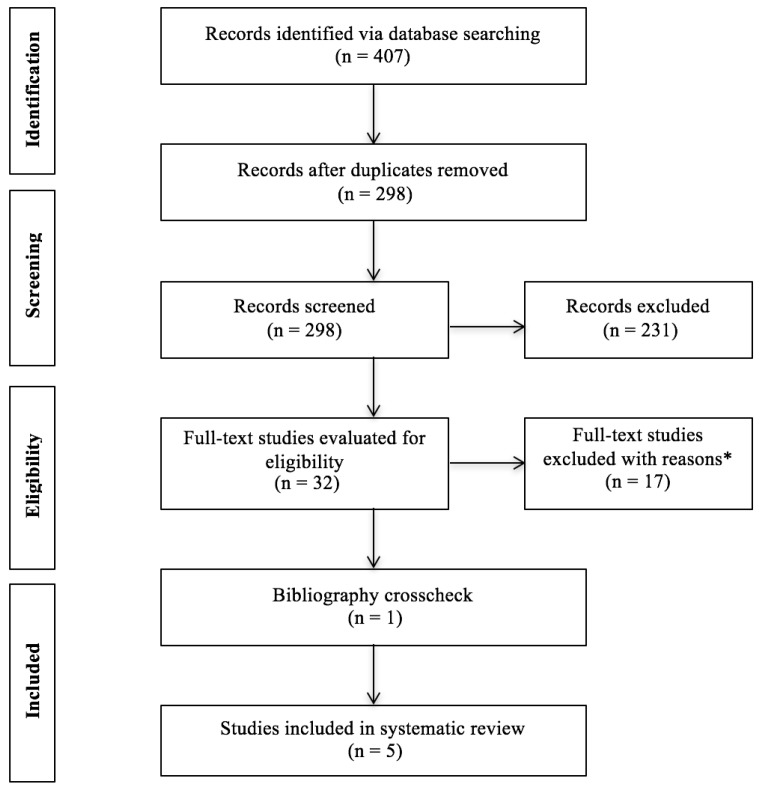
PRISMA flow diagram of studies through the review process; * reasons for study exclusion included non-English language articles, ineligible VR type (i.e., non-immersive VR), and no measures of anxiety and depression. Many studies were excluded for multiple reasons.

A total of 407 potentially relevant articles were identified. After removing duplicates, titles and abstracts of the remaining articles were screened and further identified as potentially meeting the eligibility criteria. An additional study was located through the search of reference lists. Following a thorough securitization of the full-text articles, five studies fully met the eligibility criteria and were included in this review (see Figure 1). Reasons for excluding articles included: non-English language articles, ineligible VR type (i.e., non-immersive VR), and no measures of anxiety and depression, among others. Notably, a high inter-rater agreement (97%) of the articles included was obtained between the authors.

### 3.2. Study Characteristics

The characteristics of included studies are shown in Table 2. Among the five studies, two were RCTs [24,25], one was a controlled trial (CTs; quasi-experimental pre-posttest design without randomization) [26], and two were pre-experimental designs with pre-post-test design among same participants) [27,28]. The studies were conducted in different countries: two in the U.S. [27,28], one in Brazil [25], one in Korea [24], and one in Taiwan [26], reflecting the global nature of the phenomenon under study. Among these studies, one was conducted in a clinical setting [26], but the other four were completed in a laboratory setting [24,25,27,28]. Participants’ ages ranged from 22 years to 84 years. Populations were diverse, including patients suffering from spinal-cord injuries [26], college students and faculty/staff [27,28], and older adults [24,25]. Notably, a relatively large variability in sample size and VR exposure was observed across studies, with samples varying from 30 to 154 participants (median number of participants being 70) and VR exposure length ranging from 30 min to 8 weeks (median period of exposure being 90 min). All studies examined the effects of VR exercise on health-related outcomes including physical fitness, cognitive functioning, mental health, and anxiety and depression. Measures employed to assess anxiety- and depression- related outcomes were most often the Activation-Deactivation Adjective Check List (AD-ACL) [26,27,28], Geriatric Depression Scale (GDS) [25], and Short-Form Health Survey (SF-36) [24].

### 3.3. Quality and Risk of Bias Assessment

Following the rating of each study using the 8-item quality and risk of bias assessment tool, scores ranged from 4 to 7 (see Table 1). In detail, two studies received an overall rating of high-quality/low risk of bias (scored above the median score of 4.5), while three studies received an overall rating of low-quality/high risk of bias. Notably, all studies succeeded in retaining at least 70% of the participants. The most common issues with the study quality and risk of bias were related to lack of follow-up measurements, no power calculations to determine appropriate sample sizes, and omission of discussion regarding missing data interpretation. Additionally, relevant discussions concerning potential bias and methodological efforts to minimize confounding effects were rarely mentioned within the low-quality studies. Given the poorer quality of research designs among the literature on this topic, a meta-analysis was prohibited.

### 3.4. Key Findings

Of the five studies examining the effects of VR exercise on participants’ anxiety- and depression-related outcomes, four reported significant improvements in outcomes associated with these conditions, such as reduced tiredness and tension, and increased energy and enjoyment [24,26,27,28]. Notably, one study failed to support the effectiveness of VR exercise over traditionally active exercise (without virtual reality stimulation) on depressive symptoms [25]. Specifically, Chen and colleagues [26] investigated the psychological benefits of VR exercise in patients suffering from spinal-cord injuries. An experimental group (*n* = 15, *M_age_* = 51.3, *SD* = 15.8) underwent treatment using a VR exercise bike, while a comparison group (*n* = 15, *M_age_* = 45.4, *SD* = 14.24) received an identical exercise treatment but without VR equipment. Significant differences for AD-ACL-measured calmness and tension were observed between groups, with experimental group participants experiencing the greatest reductions in tension and improvements in feelings of calmness. Additionally, Lee et al. [24] examined the effect of individualized feedback-based VR exercise on health-related quality of life in older women. Participants were randomized to either a VR exercise (motions based on Tai Chi) group (*n* = 26, *M_age_* = 68.7, *SD* = 4.6) or group-based exercise group (*n* = 28, *M_age_* = 67.7, *SD* = 4.3). Both groups received a 60-min intervention three times a week for eight weeks. The SF-36 was administered. The findings indicated that the VR exercise group not only achieved greater decreases depression compared to the group-based exercise group, but also larger improvements in quality of life, social functioning, and physical fitness.

Moreover, Plante et al. [27] conducted a cross-sectional study to assess the psychological benefits of VR-based treadmill exercise among college students (*n* = 154, 102 females). Participants were randomly assigned to one of three 20-min conditions: (1) walking on a laboratory treadmill while using VR, (2) walking on the treadmill without VR, (3) experiencing a virtual walk using VR without any exercise performed (i.e., sedentary condition), and (4) walking outside around campus. The AD-ACL was used to assess mood states, including energy, calmness, tension, and tiredness. Findings indicated that VR-based treadmill walking enhanced energy and reduced tiredness and tension to the greatest degree versus the other three groups. Similarly, Plante and colleagues [28] conducted another cross-sectional study of VR biking exercise study among University faculty and staff (*n* = 88, 44 females). Participants were randomly assigned to one of three 30-min conditions including: (1) playing a VR computer bicycle game, (2) an interactive VR bicycle experience on a computer while exercising on a stationary bike at moderate intensity, and (3) bicycling at a moderate intensity (60–70% maximum heart rate) on a stationary bicycle. The AD-ACL was administered to assess mood states before and after each exercise session, and findings favored VR-based biking exercise for the enhancement of enjoyment and energy, as well as the reduction of tiredness compared to the other two conditions.

Although a majority of the included studies (*n* = 4) supported the claim that VR exercise can improve anxiety- and depression-related outcomes, it is worth noting that one study failed to back these findings. Monteiro-Junior et al. [25] evaluated the effects of VR exercise on cognitive functions, physical performance, fear of falling, and depressive symptoms among older adults. Participants were randomly allocated to either VR-based physical exercise with exergames (*n* = 29, *M*_age_ = 85, *SD* = 8) or active control group (*n* = 41, *M*_age_ = 86, *SD* = 5), with the latter group performing same exercises without VR stimulation. Both groups received a one-week intervention lasting 30–45 min per session for 12–16 sessions. Depressive symptoms were assessed via the GDS. No significant differences between groups for depressive symptoms were observed. Notably, depressive symptoms in this study were assessed as a secondary outcome.

## 4. Discussion

The primary focus of this article was to comprehensively review the available literature regarding the effectiveness of VR exercise on anxiety and depression while attempting to provide practitioners and health professionals future directions for the use of VR exercise in treating these and other mental disorders. Five studies were included in the final analysis. Findings revealed that VR exercise had significant beneficial effects on anxiety- and depression-related outcomes in 4 of included studies. Notably, no detrimental effects were observed within any study. Overall, the present review provides initial evidence of the potential of using VR exercise to treat individuals with anxiety and depression.

The majority of the five articles included in this review indicated VR exercise to have positive effects on anxiety- and depression-related outcomes [24,26,27,28]. Although the results are encouraging, several limitations should be noted. First, relatively small sample sizes and short or unclear VR exercise exposure periods may limit the ability to generalize the findings and confirm an optimal dose (i.e., type, duration, intensity, and frequency) of VR exercise needed to treat anxiety and depression. Second, as most of the included studies were conducted in a laboratory setting (*n* = 4), integrating VR exercise into clinical practice must be viewed cautiously. Third, as technology advances, some types of VR may have been phased out by newer systems. For example, three included studies were conducted before 2010 [26,27,28]. Thus, while the VR technology used in these studies was research-grade, this technology might be antiquated compared to currently-available VR systems. These outdated VR systems may have affected the participant experience within these studies and thereby had an impact on the treatment effects. Nonetheless, the largely positive findings of this body of VR exercise literature on anxiety and depression are encouraging, as VR systems continue to improve in quality.

VR is a technology-based interface that allows users to experience computer-generated virtual environments [29]. Over the past decade, VR technology has experienced increasing application in the treatment of individuals with mental disorders [5]. As previously stated, most available VR literature focused on effects of VRET on mental disorders. VRET is based upon emotion-processing theory, which postulates that fear memories are structures and contain information regarding fear stimuli, responses, and meaning [29]. As such, VRET has been widely used in clinical research to trigger and adjust those fear structures by presenting novel incompatible information and advancing emotional processing (e.g., creating a virtual environment containing the trigger(s) of an individual’s anxiety and/or depression) [29]. Within these clinical research studies, a growing body of literature has demonstrated that VRET is related to large declines in symptoms of both anxiety and depression, and is similar in efficacy to traditional exposure therapy [13,30,31,32]. Yet, the effects of commercially-available VR exercise on mental health are rarely investigated. More research needs to be done in this area of inquiry.

Currently, only 41% of U.S. adults with a mental disorder received mental health services in the past year. This fact is concerning and suggests the need for new and enjoyable treatments for mental disorders to be included exclusively or as an adjunct to traditional treatments [33]. Fortunately, VR exercise continues to become more mainstream for health promotion and, given the demonstrated effects of exercise on anxiety and depression in addition to the enjoyable nature of VR [16,18,19], VR exercise may be a potential treatment option for these two mental disorders and others. Indeed, the latest commercially-available immersive VR headsets, such as the PlayStation VR, Samsung Gear VR, and Oculus Rift are increasingly becoming popular worldwide, presenting scholars and health professionals with opportunities to implement VR exercise interventions with the objective of promoting mental health among various populations. For instance, Zeng and colleagues (2017) [34] conducted a pilot study using a state-of-the-art VR exercise apparatus (VirZoom) and demonstrated the feasibility of using VR exercise for enhancing psychological outcomes including enjoyment and self-efficacy, indicating the potential of VR exercise technology for use in the promotion of mental health to be great. Yet, VR exercise studies akin to those of Zeng and colleagues that employ the newest VR systems among individuals with anxiety or depression, with the objective to improve the symptomology associated with these two disorders, remain to be observed.

It is noteworthy that employing the newest VR exercise systems in the promotion of psychological health among individuals with anxiety or depression offers several advantages. Specifically, the newest VR exercise systems allow for the precise presentation and control of stimuli within a dynamic multi-sensory three-dimensional computer-generated environment [35] while creating a safe and motivating exercise condition that may avoid the injury and burnout sometimes associated with exercise in a real-world setting. Given these advantages and the fact that regular exercise can have a profoundly positive impact on depression, anxiety, and other mental disorders [36,37], clinicians and health professionals should take into account the 2008 Physical Activity Guidelines for Americans [38] when considering a VR exercise prescription as an adjunctive or alternative approach to mental health treatments. As such, more research is warranted in order to determine the optimal dose of VR exercise (i.e., intensity, duration, and frequency) needed to promote the most positive psychological outcomes, which would assist in the advancement of VR exercise toward personalized medicine. Yet, if empirical research continues to demonstrate effectiveness, the use of VR exercise could offer adjunctive or alternative options for the treatment of mental disorders such as anxiety or depression.

As with any other groundbreaking innovation, VR is not devoid of potentially negative aspects. To begin, emerging evidence has suggested that the use of contemporary, consumer-oriented head-mounted display devices (e.g., Oculus Rift, Samsung Gear VR, HTC Vive, etc.) may cause motion sickness, with females at greater risk [39]. The common symptoms of motion sickness include eye strain, headache, stomach awareness, sweating, fatigue, drowsiness, disorientation, and, in some cases, nausea and vomiting [40]. Thus, VR exercise may not be a good option for those who suffer from or are at higher risk of motion sickness and live with symptoms of epilepsy such as severe dizziness, blackouts, and seizures. Further, while VR games make exercise more appealing, the equipment used for VR exercise is relatively expensive compared to traditional exercise. For example, the VirZOOM exercise bike—a virtual reality fitness game platform, which integrates a traditional stationary bike with a VR headset (e.g., PlayStation VR), costs approximately $1000 prior to purchasing compatible games. As such, the high price of VR exercise may result in potential stakeholders within the medical field adopting a “wait-and-see” approach toward the implementation of this novel technology. Additionally, as with other forms of technology, there may be some risk for VR obsession/dependence—factors that need to be considered when treating individuals with other mental disorders. Finally, while exercise has been proven to be effective in the treatment of anxiety and depression [41,42], it is worth noting that most VR exercise apparatuses are still in the exploratory phase of development and have yet to advance beyond feasibility and small case studies [43]. Therefore, VR exercise still remains widely unexplored, suggesting more research is warranted.

Although the current study’s strength lies in the provision of the first known synthesis of the effects of VR exercise on anxiety and depression in a systematic manner, the study is not without limitations. To begin, the current review only included peer-reviewed full-text and English language publications, despite the fact that other unpublished and non-English research may be available on the topic. Second, qualitative perspectives such as user experience were excluded, because they fell outside the review’s primary aim. These viewpoints, however, would have significant relevance for the treatment of mental disorders. Third, it is possible that other VR exercises exist and, as such, it is possible the search terms used in the current study limited our ability to locate all relevant studies. Finally, given a small number of empirical studies and the relatively low quality of the study designs and methodology, a conclusive statement regarding the effectiveness of VR exercise on anxiety and depression should be interpreted with caution—providing further indication of the need for greater study on this topic.

## 5. Conclusions

This preliminary review synthesizes the available experimental evidence regarding the effects of VR exercise on anxiety- and depression-related outcomes. Findings favor VR exercise, as this small group of studies indicates improvements in mental health for those who had these two mental disorders. Yet, the paucity of literature on this topic and the need for higher-quality study designs among large samples necessitates further research prior to large-scale implementation of VR exercise treatments for anxiety and depression within clinical settings.

## Figures and Tables

**Table 1:** Design Quality Analysis.

Articles	Randomization	Control	Pre-Post	Retention	Missing Data	Power Analysis	Validity Measure	Follow-Up	Score	Effectiveness
Lee et al. [24]	+	+	+	+	−	−	+	+	6	Yes
Monteiro-Junior et al. [25]	+	+	+	+	−	+	+	+	7	No
Chen et al. [26]	−	+	+	+	−	−	+	−	4	Yes
Plante et al. [27]	+	−	+	+	−	−	+	−	4	Yes
Plante et al. [28]	+	−	+	+	−	−	+	−	4	Yes

Note: “+” refers to positive (explicitly described and present in details); “−” refers to negative (inadequately described and absent); “Yes” indicates significant positive effect; ‘’No’’ indicates no significant effect; median score = 4.5.

**Table 2:** Descriptive Characteristics of Included Studies.

Study Description	Design/Sample	Type	Outcomes/Instrument	Exposure	Duration	Findings
Chen et al. [26] 2009, Taiwan	Quasi-experiment; *N* = 30 Patients suffering from spinal-cord injury; Intervention (*n* = 15, *M_age_* = 51.3, *SD* = 15.8); Control (*n* = 15, *M_age_* = 45.4, *SD* = 14.24)	VR-based exercise bike	Mood states were assessed via Activation–Deactivation Adjective Check List (AD-ACL)	Rehabilitation therapy with a VR-based exercise bike Vs. same therapy without VR	Not applicable	A virtual-reality-based rehabilitation program can ease patients’ tension and induce calm
Lee et al. [24] 2015, Korea	Randomized controlled trial (RCT); *N* = 54; Intervention (*n* = 26, *M_age_* = 68.7, *SD* = 4.6); Control (*n* = 28, *M_age_* = 67.7, *SD* = 4.3)	Individualized feedback-based VR exercise	Psychological outcomes were measured via Short-Form Health Survey (SF-36)	Individualized feedback-based VR exercise Vs. group-based exercise	a 60-min intervention three times a week for eight weeks.	Individualized feedback-based virtual reality group (IFVRG) showed greater improvement in mental health (increased social functioning and decreased depression)
Monteiro-Junior et al. [25] 2017, Brazil	RCT; *N* = 70 older adults; Intervention (*n* = 29, *M*_age_ = 85, *SD* = 8); Control (*n* = 41, *M*_age_ = 86, *SD* = 5)	VR-based physical exercise	Depressive symptoms were assessed via Geriatric Depression Scale (GDS)	Exercises with VR stimulation Vs. same exercises without VR stimulation	30–45 min/session, 12–16 sessions twice a week	There was no significant difference between groups in depressive symptoms
Plante et al. [27] 2003, USA	Cross-sectional; *N* = 154 (102 females) students were assigned to 4 different conditions	VR with walking on treadmill	Mood states were assessed via AD-ACL, including energy, calmness, tension, and tiredness	Brisk outdoor walk Vs. VR with walking on treadmill Vs. Walking on the treadmill without VR Vs. VR without exercise	4 × 20-min experiments	VR may enhance energy and reduce tiredness and tension when paired with actual exercise
Plante et al. [28] 2003, USA	Cross-sectional; *N* = 88 (44 females, *M_age_* = 38.1, *SD* = 12.31) university faculty and staff s were assigned to 3 different conditions	VR in combination with stationary bike	Mood states were assessed via AD-ACL, including energy, calmness, tension, and tiredness	Stationary bicycling at a moderate intensity (60–70% maximum heart rate) Vs. VR-based bicycle game without actual exercise Vs. (3) VR-based stationary bike at moderate intensity	3 × 30-min experiments	VR when paired with exercise enhances enjoyment, energy, and reduces tiredness. Notably, VR without exercise was found to increase tension, tiredness, and lower energy level
